# Generation**** of Pure Highly Functional Human Anti-Tumor Specific Cytotoxic T Lymphocytes With Stem Cell-Like Memory Features for Melanoma Immunotherapy

**DOI:** 10.3389/fimmu.2021.674276

**Published:** 2021-09-08

**Authors:** Mohamad Hamieh, Jean-François Chatillon, Estelle Dupel, Florence Bayeux, Emilie Fauquembergue, Pauline Maby, Aurelie Drouet, Anne-Bénédicte Duval-Modeste, Sahil Adriouch, Olivier Boyer, Jean-Baptiste Latouche

**Affiliations:** ^1^Normandie University, UNIROUEN, Inserm U1245, Institute for Research and Innovation in Biomedecine (IRIB), Rouen, France; ^2^Normandie University, UNIROUEN, Inserm U1234 - Pathophysiology, Autoimmunity, Neuromuscular diseases and regenerative THERapies (PANTHER), IRIB, Rouen, France; ^3^Department of Dermatology, Rouen University Hospital, Rouen, France; ^4^Department of Immunology and Biotherapy, Rouen University Hospital, Rouen, France; ^5^Department of Genetics, Rouen University Hospital, Rouen, France

**Keywords:** adoptive cell therapy, MART-1/Melan-A, anti-tumor cytotoxic T lymphocytes, stem cell-like memory T cells, artificial antigen presenting cells

## Abstract

Adoptive immunotherapy based on the transfer of anti-tumor cytotoxic T lymphocytes (CTLs) is a promising strategy to cure cancers. However, rapid expansion of numerous highly functional CTLs with long-lived features remains a challenge. Here, we constructed NIH/3T3 mouse fibroblast-based artificial antigen presenting cells (AAPCs) and precisely evaluated their ability to circumvent this difficulty. These AAPCs stably express the essential molecules involved in CTL activation in the HLA-A*0201 context and an immunogenic HLA-A*0201 restricted analogue peptide derived from MART-1, an auto-antigen overexpressed in melanoma. Using these AAPCs and pentamer-based magnetic bead-sorting, we defined, in a preclinical setting, the optimal conditions to expand pure MART-1-specific CTLs. Numerous highly purified MART-1-specific CTLs were rapidly obtained from healthy donors and melanoma patients. Both TCR repertoire and CDR3 sequence analyses revealed that MART-1-specific CTL responses were similar to those reported in the literature and obtained with autologous or allogeneic presenting cells. These MART-1-specific CTLs were highly cytotoxic against HLA-A*0201^+^ MART-1^+^ tumor cells. Moreover, they harbored a suitable phenotype for immunotherapy, with effector memory, central memory and, most importantly, stem cell-like memory T cell features. Notably, the cells harboring stem cell-like memory phenotype features were capable of self-renewal and of differentiation into potent effector anti-tumor T cells. These “off-the-shelf” AAPCs represent a unique tool to rapidly and easily expand large numbers of long-lived highly functional pure specific CTLs with stem cell-like memory T cell properties, for the development of efficient adoptive immunotherapy strategies against cancers.

## Introduction

Anti-CD19 CAR (Chimeric Antigen Receptor-modified)-T cells have recently encountered impressive therapeutic successes in malignant CD19^+^ hemopathies. They underline the potential clinical interest of being able of amplifying *in vitro* “natural” anti-tumor T lymphocytes (TLs) for adoptive cell therapy (ACT), especially in solid tumors such as melanoma (1). Specific *in vitro* T lymphocyte (TL) activation for ACT requires efficacious antigen presentation. Antigen presenting cells (APCs) deliver three essential signals to TLs, the first one being provided by specific MHC-peptide complexes interacting with T cell receptors (TCRs), the second one by accessory molecules, and the third one by cytokines and chemokines secreted in the T cell environment. To provide these three signals *in vitro*, Peripheral Blood Mononuclear Cell (PBMC) feeder cells, virus-transformed or CD40-activated B cells and monocyte-derived dendritic cells can be used as APCs (2–7). TL activation with anti-CD3 and anti-CD28 antibodies and high doses of IL-2 can also be envisaged (8, 9). Many of these approaches have already been used in different clinical trials. Nevertheless, these techniques require long and non-standardized procedures. Obtaining TLs in a sufficient number with suitable phenotype and function for successful immunotherapy remains difficult.

To circumvent these difficulties, different groups have thus developed standardized, “off-the-shelf” artificial antigen presenting systems to deliver the appropriate signals to rapidly generate *in vitro* anti-tumor TLs (10). With this aim, beads, allogeneic or xenogeneic cells have been used (11–15). Different works have already given hints that artificial antigen presenting systems based on beads complexed with HLA molecules or anti-CD3/anti-CD28 antibodies have lower capacities to activate TLs than more physiological systems such as cell-based systems (10, 16). Among the different cell-based systems, human K562-based artificial antigen presenting cells (AAPCs) have been successfully used in phase I clinical trials in melanoma and are still optimized (14, 17). Noteworthy, K562 cells expressing a truncated form of CD19 for expanding *in vitro* anti-CD19 CAR-T cells did not show superiority to conventional protocols in a phase I clinical trial (18) and most of the cell-based artificial antigen presenting systems are still in preclinical development (19).

Many artificial TL activation systems mentioned above lead to the expansion of some TLs which are not cells of therapeutic interest. For example, CAR-T cells expanded *in vitro* with anti-CD3/anti-CD28 beads are injected without further purification, along with autologous activated T cells of unknown specificity. The presence of non-specific TLs could decrease ACT efficiency and eventually generate side effects. This problem has been circumvented with either multiple stimulations to obtain a more specific response or the injection of tumor specific-derived TL clones (20–22). However, numerous *in vitro* TL stimulations and/or *in vitro* selected clones without an appropriate phenotype could lead to impaired reactivity *in vivo* (23). The common source of TLs which are used in the different studies are tumor infiltrating lymphocytes (TILs), TLs from patients’ draining lymph nodes (LNs) or PBMCs (2–4, 15, 20). Many difficulties are associated with TIL and LN manipulation, because they mainly contain terminally differentiated TL effectors, which have been shown to display deficient function, impaired proliferation and survival *in vitro* (24, 25). Consequently, PBMCs were suggested as an interesting source of anti-tumor TLs, even as good, at least, as the tumor itself (4, 15, 17). A new idea has thus emerged that “younger”, less differentiated TLs with long-lived phenotype would be better tools for ACT (26, 27). ACT protocols had already proven that central memory TLs (T_CM_) displayed more interesting anti-tumor capacities than effector memory TLs (T_EM_) (27, 28). More recently, in mice, non-human primates and humans, a subpopulation of memory TLs called stem cell-like memory TLs (T_SCM_), which is less differentiated than T_CM_, was characterized. They express in humans different common markers found on naïve TLs (including CD45RA and CD62L) and a marker of already activated TLs, CD95, thus making them more differentiated than naïve TLs. Moreover, they display high proliferation, self-renewal, long-life and multipotency features, giving rise to all the other memory subsets (29–33).

However, obtaining anti-tumor TLs displaying T_SCM_ features in an easy and reproducible manner in an *in vitro* protocol has not been reported yet. Recent works have proposed using anti-CD3/anti-CD28-based protocol to activate naïve TLs from peripheral blood in CMV infection and graft versus host disease (GVHD) contexts (31, 32). Nevertheless, a protocol allowing the generation of tumor-specific TLs displaying T_SCM_ features has not been proposed yet (34).

In this study, we used AAPCs generated in our laboratory, derived from murine NIH/3T3 fibroblasts and expressing the most frequent HLA class I molecule, HLA-A*0201, the three main accessory molecules (CD54, CD58, CD80) and a MART-1-derived peptide, MART-1 being an overexpressed antigen in melanoma (15). We thus designed an AAPC-based preclinical procedure starting from peripheral blood TLs (PBTLs) as a source of specific TLs to obtain melanoma-specific CTLs usable for ACT. We obtained a pure population of MART-1-specific CTLs (M1-CTLs) in sufficient numbers for ACT. Phenotypic study revealed that these highly functional M1-CTLs harbored long-lived, renewable and non-exhausted T cell features. Therefore, in this preclinical study, by using our AAPC system, we propose a protocol which favors the generation of pure tumor-specific TLs displaying T_EM_, T_CM_ and T_SCM_ features which could prove very promising for an efficacious anti-tumor ACT protocol.

## Materials and Methods

### Recruitment of Healthy Donors and Melanoma Patients

Ten healthy donors and six grade II/III melanoma patients were recruited based on Human Leukocyte Antigen (HLA)-A2 expression assessed by flow cytometry. Healthy donors and patients were enlightened and signed informed consent under agreement from Rouen University Hospital Institutional Review Board “Comité de Protection des Personnes Nord-Ouest I” which approved this study.

### Construction of NIH/3T3-Derived Artificial Antigen-Presenting Cells

Vector construction and gene transfer procedures to generate NIH/3T3 mouse fibroblast (ATCC, Manassas, VA)-derived AAPCs cultured in DMEM-10% DCS (Donor Calf Serum, ThermoFisher Scientific, Illkirch, France) were previously described (15, 35). They stably express the human HLA-A*0201 heavy chain, β-2-microglobulin, B7.1 (CD80), ICAM-1 (CD54) and LFA-3 (CD58) molecules, as well as MART-1_A27L_ analogue peptide (M1m, ELAGIGILTV), described as more immunogenic than the native MART-1 peptide (M1, 34).

Both the expression of transgenes and the absence of mycoplasma contamination were ascertained as described (35) and by regular PCR and Hoechst staining, respectively, every three months.

### Purification of T Lymphocytes and Co-Culture With AAPCs

PBMCs were collected by density centrifugation on a lymphocyte separation medium (Eurobio, Courtaboeuf, France). Irradiated AAPCs (10^5^ cells per well, 25 Gy) were plated in 24-well plates in AIM-V medium (Invitrogen, Saint Aubin, France) supplemented with 5% DCS. The next day, total TLs were negatively selected from PBMCs using Dynabeads untouched human T cell Kit (Invitrogen) according to manufacturer’s protocol.

Purified TLs (10^6^ cells per well) resuspended in AIM-V medium were co-cultured with irradiated AAPCs at a ratio of 10 to 1 for 21 days as previously described (15, 35). On day 7 of the co-culture and then, every third day, 20 IU/ml of IL-2 (R&D systems, Abington, UK) were added to the wells.

At D21, following manufacturers’ protocols, phyco-erythrin (PE)-labeled Pro5 MHC class I Pentamers (PentM1m, Proimmune, Oxford, UK) with anti-PE microbeads (Miltenyi Biotec, Paris, France) were used to magnetically sort M1-CTLs from obtained TLs. During the different steps of purification, cells and sorting buffer (PBS, 0.1% Bovine Serum Albumin (BSA, Sigma-Aldrich, Lyon, France) and 2 mM EDTA (VWR, Fontenay-sous-Bois, France) were always kept on ice. Purified TLs (0.5x10^6^) were then amplified on 10^5^ irradiated AAPCs per well in 24-well plates for an additional 14 days as described above except that IL-2 was first added 2h after co-culture start.

### Immunoscope

M1-CTLs obtained at day 35 were harvested and CD8^+^ TLs were purified using human CD8 microbeads (Miltenyi Biotec) according to manufacturer’s protocol. CD8^+^ TL and M1-CTL RNA were isolated using GenElute Mammalian Total RNA miniprep kit (Sigma-Aldrich). Complementary DNA (cDNA) was obtained by RNA reverse transcription using M-MLV reverse transcriptase (Promega, Charbonnière, France) following manufacturer’s protocol. Precise procedures and primer sequences to study complementary determining region 3 (CDR3) were described previously (36). PCR products were examined in an Applied Biosystem/Hitachi 3100 genetic analyzer (Hitachi Electronics Engineering, Tokyo, Japan). Data were analyzed with Sequencing Analysis software (Invitrogen).

### Flow Cytometry Reagents and Analyses

Pentamers (Pent) complexed with M1m peptide (PentM1m) or control Flu Matrix Protein-derived peptide (FMP, PentFMP), labeled with PE or allophycocyanine (APC) molecule (Proimmune), were used as recommended by the manufacturer. Anti-CD8 labeled with either fluorescein isothiocyanate (FITC, Invitrogen), or peridinin-chlorophyll-protein (PerCP) or APC (eBiosciences, Paris, France), anti-CD4-PE, anti-CD14-FITC, anti-CD16-FITC, anti-CD19-PE, anti-CD54-PE, anti-CD56-PE, anti-CD80-FITC (Invitrogen), anti-CD27-APC-efluor780 or anti-CD27-APC-efluor450, anti-CD28-PE-Cy7, anti-CD44-PerCP-Cy5.5, anti-CD45RA-FITC or -PECy7, anti-CD45RO-PerCP-efluor710, anti-CD57-FITC, anti-CD62L-APC-efluor780, anti-CD95-FITC, anti-CD122-PE, anti-CD127-FITC, anti-CTLA-4-PE, anti-FasL-PE, anti-CCR7-PE, anti-PD-1-PerCP-efluor710 (eBiosciences), anti-CD58-FITC, anti-β2-microglobulin-PE, anti-HLA-A2-FITC, anti-BTLA-PE, anti-CLA-FITC, anti-CCR4-PE (BD Biosciences, Le Pont de Claix, France) and anti-CCR10-APC (R&D systems) antibodies were used with corresponding isotypic controls.

IOTest Beta Mark TCR Vβ Repertoire Kit (Beckman Coulter, Villepinte, France) was used to study 24 Vβ subfamilies following manufacturer’s instructions.

For intracellular study, cells were incubated during 4h with either AAPCs as target cells or phorbol myristate acetate (PMA, 1 µg/ml)/ionomycin (10 µg/ml, Sigma-Aldrich), both in the presence of brefeldin A (20 µg/ml, Sigma-Aldrich), then labeled with anti-CD8-APC for 30 min at 4°C, fixed and permeabilized using Intraprep kit (Beckman Coulter) following manufacturer’s instructions. Anti-IL-2-FITC, anti-IFNγ-PerCP, anti-TNFα-PE (Immunotech, Marseille, France) antibodies were used following manufacturer’s instructions. Bcl-2 expression was assessed using Intraprep kit and Bcl-2 FITC kit (BD Biosciences) following manufacturer’s recommendations.

All cells were resuspended in sorting buffer. Flow cytometry measurements were performed on FACSCanto and BD LSRFortessa systems (BD Biosciences) and analyses were made using FlowJo software (TreeStar Inc., OR).

### Memory TL Sorting

Cell sorting was performed using a FACSAria cell sorter (BD Biosciences). At day 21, TLs were first labeled as described above with the following reagents: PentM1m-APC and CD45RA-FITC, CD62L-APC-efluor780 antibodies. PentM1m^+^CD45RA^+^CD62L^+^ or CD62L^-^ CTLs were sorted reaching a purity always greater than 98%. Sorted TLs (0.5x10^6^) were co-cultured with 10^5^ irradiated AAPCs per well in 24-well plates for 14 days as described above in AIM-V medium supplemented with penicillin (50IU/ml), streptomycin (50µg/ml) and gentamicin (50 µg/ml, Invitrogen).

### CTL Cytotoxicity and Functional Avidity Studies

Standard Chromium (^51^Cr) release assays were performed as already described (35), using the following cell lines: TAP-deficient HLA-A*0201^+^ T2 (ATCC), MART-1^+^HLA-A*0201^+^ melanoma-derived M102, M102 cells transduced to overexpress MART-1 (M102-M1), MART-1^+^HLA-A*0201^-^ M140 and MART-1^-^HLA-A*0201^+^ R104 (M102, M140 and R104 cell lines were kind gifts from Prof. F. Jotereau’s team, Inserm U892, Nantes, France). All cell lines were labeled for 1h at 37°C with ^51^Cr (PerkinElmer, Courtaboeuf, France). T2 cells were then pulsed with a relevant or an irrelevant peptide (respectively M1, AAGIGILTV, M1m, ELAGIGILTV or FMP, GILGFVFTL, synthesized in the Inserm unit U413, Rouen, France) for 1h at room temperature.

For cytotoxicity assays, the final peptide concentration of 10 µM was used. Five thousand target cells were incubated with TLs at different ratios during 4h at 37°C.

For functional avidity assays, peptide concentrations ranging from 10 to 10^-6^ µM were used. Five thousand T2 cells were incubated with TLs at a unique ratio of 10 to 1 during 4h at 37°C.

Expression of relevant antigens (HLA-A2, MART-1) by all cell lines and the absence of mycoplasma contamination were ascertained as described (35) and by regular PCR and Hoechst staining, respectively, every three months.

### Statistical Analysis

We used Prism software (GraphPad software Inc, La Jolla, CA) in order to perform two way-unpaired *t* tests or Friedman tests with Dunn’s post-tests. All error bars represent standard errors of the means (SEMs). ns (not significant) was used for *p*>0.05, * for *p*<0.05, ** for *p*<0.01.

## Results

### Rapid Generation of Pure MART-1-Specific Cytotoxic T Lymphocytes Using a NIH/3T3-Derived Artificial Antigen Presenting Cell-Based Protocol

**Figure 1:**
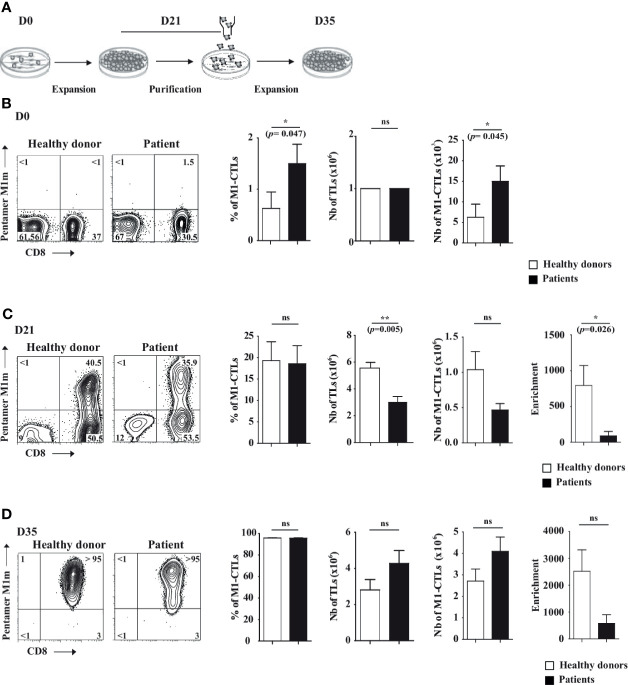
AAPC-based protocol to obtain pure MART-1-specific cytotoxic T lymphocytes. **(A)** Scheme describing the NIH/3T3-derived AAPC-based protocol in three steps: first, at day **(D)** 0 of the co-culture, total TLs were co-cultured with irradiated AAPC^M1m^. At D21, MART-1-specific cytotoxic TLs (M1-CTLs) were purified by magnetic sorting and expanded until D35. **(B–D)** At D0 **(B)**, D21 **(C)** and D35 **(D)**, MART-1 expression was assessed using PE-labeled Pentamer and APC-labeled anti-CD8 (left parts). Histograms of M1-CTL percentages, TL absolute numbers and M1-CTL absolute numbers respectively (right parts) are displayed. Left parts. Representative examples of patients and healthy donors are displayed. Quadrants are placed according to isotype control staining. Relative percentages are shown in each quadrant. Right parts. Histograms were obtained from ten healthy donors (white bars) and six patients (black bars) and shown with standard errors of means. Statistical tests (*t* test) were performed to compare both groups. Not significant (ns): *p* > 0.05, *p < 0.05, **p < 0.005 (p=0.0048).

NIH/3T3-derived AAPCs have been described as an efficient tool to expand antigen-specific CTLs (15). AAPCs reconstitute a human HLA-A*0201-restricted immunological synapse, including accessory molecules CD54, CD58, CD80 and present for this study MART-1_A27L_ analogue peptide on HLA-A*0201 molecule (AAPC^M1m^). Total TLs were magnetically isolated with more than 95% of purity from PBMCs (data not shown) and co-cultured with AAPC^M1m^ (day 0 (D0), **Figure 1A**). At each step of our protocol, M1-CTL percentage was assessed by flow cytometry (**Figures 1B–D**). After one round of expansion, M1-CTLs were purified based on a PE-labeled Pentamer staining followed by incubation with anti-PE magnetic bead sorting (day 21 (D21), **Figure 1A**). Purified M1-CTLs were finally co-cultured with AAPC^M1m^ to expand them until day 35 (D35, **Figures 1A, D**).

At D0, 10^6^ TLs per ml were deposited on AAPC^M1m^ (**Figure 1A**). Total TLs from patients and healthy donors contained in average around 1.5% and 0.5% of M1-CTLs respectively, representing around 15x10^3^ and 5x10^3^ M1-CTLs per million TLs at D0 (**Figure 1B**). Both M1-CTL percentages and absolute numbers at D0 were significantly higher in patients than in healthy donors (*p*=0.047 and *p*=0.045 respectively, **Figure 1B**).

At D21, we obtained in average around 3x10^6^ and 5.6x10^6^ TLs in patients and healthy donors respectively, indicating a significantly lower number of TLs in patients (*p*=0.0048, **Figure 1C**). Obtained TLs contained around 19% of M1-CTLs both in patients and in healthy donors (**Figure 1C**). Although no significant differences in M1-CTL percentages and absolute numbers were found between patients and healthy donors, a significantly lower enrichment in M1-CTL population was observed in patients compared to healthy donors (*p*=0.026, **Figure 1C**) at D21.

At D35, at the end of our protocol, we obtained around 4.3x10^6^ and 2.8x10^6^ TLs in patients and healthy donors respectively (**Figure 1D**). Obtained TLs contained around 96% of M1-CTLs both in patients and in healthy donors (**Figure 1D**). No significant differences in M1-CTL percentages and absolute numbers were found between patients and healthy donors (**Figure 1D**).

With our protocol, between the initial (D0) and the final step (D35), M1-CTL number fold enrichment was in average around 600 and 2500 in patients and healthy donors respectively, although no significant differences could be found anymore between both groups because of large inter-individual fold number variability (**Table 1** and **Figure 1D**) at D35.

**Table 1:** Increase in M1-CTLs with the AAPC-based protocol.

	D0			D21			D35			Enrichment
	Nb of TLs (x10^6^)	% of M1-CTLs	Nb of M1-CTLs (x10^3^)	Nb of TLs (x10^6^)	% of M1-CTLs	Nb of M1-CTLs (x10^6^)	Nb of TLs (x10^6^)	% of M1-CTLs	Nb of M1-CTLs (x10^6^)	
D1	1.0	≤ 0.1	≤ 1.0	3.0	20.0	0.6	1.9	95.0	1.8	≥1800.0
D2	1.0	≤ 0.1	≤ 1.0	5.3	4.0	0.2	0.52	95.0	0.48	≥480.0
D3	1.0	≤ 0.1	≤ 1.0	6.0	25.0	1.5	2.5	95.0	2.4	≥2400.0
D4	1.0	≤ 0.1	≤ 1.0	6.6	35.0	2.3	7.3	95.0	6.9	≥6900.0
D5	1.0	≤ 0.1	≤ 1.0	5.2	4.0	0.2	2.14	97.0	2.08	≥2080.0
D6	1.0	≤ 0.1	≤ 1.0	5.5	40.0	2.2	6.9	95.0	6.6	≥6600.0
D7	1.0	3.2	32.0	4.0	31.2	1.3	14.7	97.3	14.3	446.9
D8	1.0	0.2	2.2	5.9	23.2	1.4	9.0	96.0	8.7	3945.5
D9	1.0	1.1	11.0	6.5	8.2	0.5	3.6	97.2	3.5	318.2
D10	1.0	1.2	12.0	7.6	2.3	0.2	2.5	95.0	2.4	196.7
P1	1.0	3.0	30.0	4.4	17.0	0.8	3.7	96.0	3.5	117.3
P2	1.0	1.5	15.0	1.4	35.9	0.5	5.4	95.9	5.2	346.7
P3	1.0	1.5	15.0	2.8	19.0	0.5	6.4	95.0	6.0	400.0
P4	1.0	1.3	13.0	2.8	19.0	0.5	5.6	96.0	5.4	415.4
P5	1.0	1.6	16.0	4.0	3.5	0.1	0.65	95.0	0.62	38.8
P6	1.0	≤ 0.1	≤ 1.0	2.6	17.0	0.4	2.3	96.0	2.2	≥2160.0

T lymphocytes from ten healthy donors (D1-10) and six grade II/III melanoma patients (P1-6) were co-cultured with AAPC^M1m^. TL absolute number, M1-CTL percentage and M1-CTL absolute number were assessed using PE-labeled Pentamer complexes at D0 (0.1% of M1-CTLs being at the sensitivity and specificity limit conservatively fixed for these tests), after one stimulation (D21) and after purification followed by a second stimulation on AAPC^M1m^ (D35). All numbers are normalized to a starting number of 10^6^ TLs at D0. M1-CTL enrichment was calculated with the following formula: number of M1-CTLs at day 35/number of M1-CTLs at day 0.

### Oligoclonal Expansion Restricted to Several Vβ Subfamilies of Obtained Pure M1-CTLs

To characterize TCR usage in obtained M1-CTLs, we analyzed TL repertoire by flow cytometry and immunoscope.

**Figure 2:**
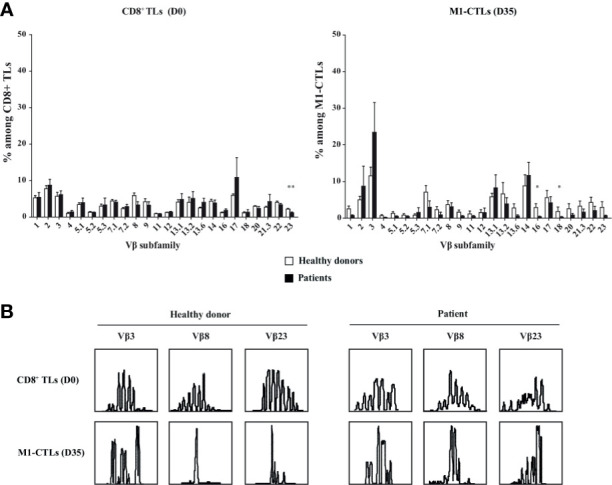
M1-CTL repertoire displays an oligoclonal response and an expansion restricted to some Vβ subfamilies. **(A)** Histograms of the Vβ subfamily relative percentages on purified CD8^+^ TLs at D0 and on M1-CTLs at D35, using FITC-, PE- or FITC-PE-labeled antibodies are displayed. Histograms were obtained from ten healthy donors (white bars) and six patients (black bars) and shown with standard errors of means. Statistical tests (*t* test) were performed to compare both groups. *p < 0.05, **p < 0.01. **(B)** Two representative examples of immunoscopes obtained with purified CD8^+^ TLs at D0 (upper panels) and with M1-CTLs at D35 (lower panels) for a healthy donor (left panels) and a patient (right panels) are represented. For both groups, representative examples of expanded (Vβ3), stable (Vβ8) and decreased (Vβ23) populations respectively are displayed.

First, we studied obtained TLs with antibodies specific of 24 different T cell receptor (TCR) beta chain variable subfamilies (Vβ). We observed a large distribution of TLs in the different Vβ subfamilies (**Figure 2A**, left panel). However, Vβ2, 3, 7.1, 13.1, 13.2, 14, 17 and 22 subfamilies preferentially expanded with our protocol (**Figure 2A**, right panel).

Then, analysis of CDR3 length using immunoscope showed that all Vβ subfamilies displayed an almost normal distribution in purified CD8^+^ TLs at D0 (**Figure 2B**, upper panels) with variations in and between both patients and healthy donors. At D35, all Vβ families displayed skewed patterns in M1-CTLs (**Figure 2B**, lower panels), with the selection of numerous different clones.

### Highly Cytotoxic Function of Obtained Pure M1-CTLs

**Figure 3:**
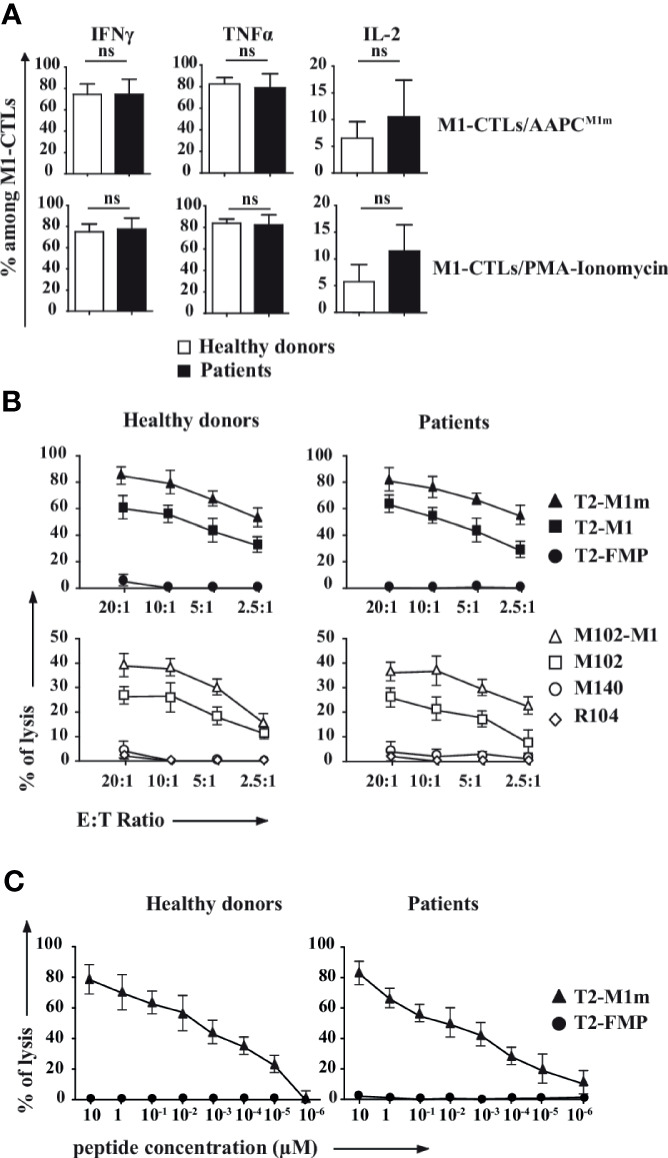
M1-CTLs secrete high levels of cytotoxicity-associated molecules, and display both high cytotoxicity and functional avidity. **(A)** Expression of IFNγ, TNFα and IL-2 in M1-CTLs at D35 was assessed by flow cytometry in the presence of either AAPC^M1m^ (upper panels) or PMA (1µg/ml)/ionomycin (10µg/ml, lower panels), both in the presence of brefeldin A (20µg/ml) for 4h. Histograms were obtained from ten healthy donors (white bars) and six patients (black bars) and shown with standard errors of means. Statistical tests (*t* tests) were performed to compare both groups. Not significant (ns): *p* > 0.05. **(B)** M1-CTL cytotoxic specific activity was assessed in standard 4-hour ^51^Cr release assays. Target cells were T2 cells (upper graphs) pulsed with MART-1_A27L_ analogue peptide (M1m, squares), MART-1 native peptide (M1, triangles), or Flu Matrix Protein-derived control peptide (FMP, circles) at a peptide concentration of 10µM. Melanoma cell lines (lower graphs) HLA-A*0201^+^MART-1^+^ (M102, open squares), transfected to overexpress MART-1 (M102-M1, open triangles) were also used as target cells. HLA-A*0201^-^MART-1^+^ (M140, open circles) and HLA-A*0201^+^MART-1^-^ (R104, open diamonds) cells were used as controls. Ratios of effector cells (E) per target cells (T) 20 to 1, 10 to 1, 5 to 1 and 2.5 to 1 were used in our experiments. **(C)** M1-CTL avidity assay was performed using T2 cells pulsed with various concentrations (from 10 to 10^-6^ µM) of MART-1_A27L_ analogue peptide (M1m) as target cells. FMP was used as a control for non-specific lysis. **(B, C)** Graphs represent the results obtained with ten healthy donors (left graphs) and six patients (right graphs), each donor having been studied in three independent experiments. Data are represented with standard errors of means.

In order to evaluate the cytotoxic potential of obtained M1-CTLs we first assessed that M1-CTLs secreted IFNγ, TNFα and IL-2. With no significant difference between patients and healthy donors, we found that around 80% of obtained TLs were high producers of IFNγ and TNFα (**Figure 3A**, left and middle panels), while IL-2 production in our conditions was limited to around 10% of obtained TLs with donor-dependent variations (**Figure 3A**, right panel).

We then verified that M1-CTLs were able to specifically and efficaciously kill T2 cells pulsed with MART-1 native peptide (M1) and, more efficiently, T2 cells pulsed with MART-1 analogue peptide (M1m) in a Chromium release assay (**Figure 3B**, upper panels). M1-CTLs were also able to efficiently lyse HLA-A*0201^+^MART-1^+^ melanoma cell lines (M102) at all tested ratios, with increased cytolysis for MART-1-overexpressing modified melanoma cell line (M102-M1, **Figure 3B**, lower panels).

Finally, using a functional avidity assay on T2 cells pulsed with decreasing concentrations of M1m peptide, from 10 to 10^-6^ µM, we observed that M1-CTLs had similar functional avidity in patients and healthy donors with still measurable lysis at peptide concentrations of 10^-5^ µM and even 10^-6^ µM for patients (**Figure 3C**).

Therefore, M1-CTLs obtained with our protocol displayed similar high cytotoxic molecule secretion and high cytotoxic function in both patients and healthy donors.

### Suitable Phenotype for Immunotherapy of Obtained Pure M1-CTLs

**Figure 4:**
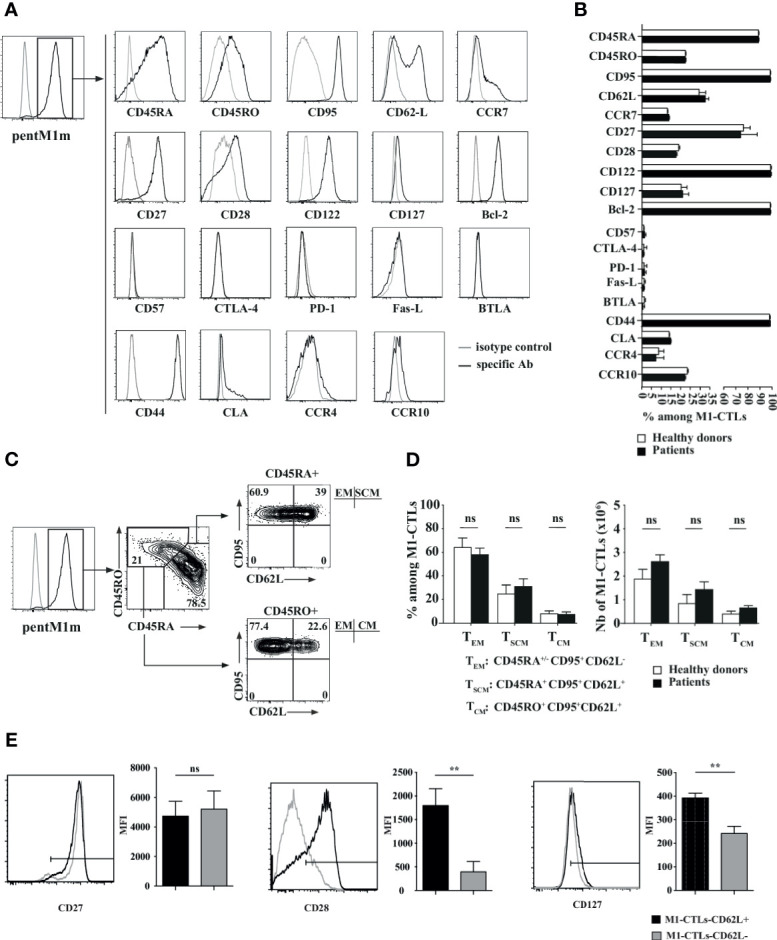
Phenotypic study of pure M1-CTLs at D35 reveals the presence of central memory, effector memory and stem cell-like memory T lymphocytes. **(A)** Representative examples of expression profiles for different families of cell surface markers on PentM1m-stained M1-CTLs obtained at D35. Cell surface markers are distributed as follows (from upper to lower graphs): differentiation state markers, activation state markers, functional inhibition markers and homing markers. Isotype control (grey line) and specific staining (black line) for the respective markers studied are shown. **(B)** Percentages of M1-CTLs positive for the cell surface markers represented in histograms. **(C)** M1-CTL CD62L/CD95 flow cytometric analyses shown for the CD45RA^+^ (upper right panel) and the CD45RO^+^ (lower right panel) fractions. Three memory T-cell subsets were defined according to the expression of CD45RA, CD45RO, CD95 and CD62L: effector memory (EM), stem cell-like memory (SCM) and central memory (CM) TLs. Relative percentages from a representative experiment are shown for each gate and quadrant. **(D)** M1-CTL percentages (left panel) and absolute numbers (right panel) of the three defined memory T-cell subsets (T_EM_, T_SCM_ and T_CM_) represented in histograms. **(B, D)** Histograms were obtained from ten healthy donors (white bars) and six patients (black bars). **(E)** Comparison of CD27, CD28 and CD127 mean fluorescence intensity (MFI) staining of CD62L^+^ gated M1-CTLs (black profiles and bars) and CD62L^-^ gated M1-CTLs (gray profiles and bars). Histograms were obtained from six healthy donors. Data are shown with standard errors of means. Statistical tests (t tests) were performed to compare both groups. Not significant (ns): *p* > 0.05, **p < 0.005.

In order to study the phenotype of obtained M1-CTLs, we performed a flow cytometry phenotypic study on M1-CTLs obtained at the end of our protocol (D35). We found that all obtained M1-CTLs expressed CD95 and that, among them, more than 85% TLs were positive for CD45RA (**Figures 4A, B**). CD45RO was expressed by around 20% of all M1-CTLs, 80% of these CD45RO^+^ cells expressing both CD45RO and CD45RA. Approximately 30% of all M1-CTLs expressed CD62L and 10% co-expressed CD62L and CCR7 (**Figures 4A, B**, and data not shown).

All obtained M1-CTLs both in patients and healthy donors expressed CD44, CD122 and Bcl-2. A majority of them, around 75%, expressed CD27. CD28 and CD127 were expressed by around 25% of obtained M1-CTLs. In addition, almost no obtained TLs expressed final differentiation and inhibition markers CD57, CTLA-4, PD-1, FasL and BTLA. Moreover, part of obtained M1-CTLs (15 to 20%) expressed the skin homing factors CLA, CCR4 and CCR10 (**Figures 4A, B**). For all of these phenotypic markers, no significant difference was observed between healthy donors and patients.

Depending on the expression of CD45RA and CD62L, we defined TLs with stem cell memory (T_SCM_), central memory (T_CM_) and effector memory (T_EM_) features in M1-CTLs (**Figures 4C, D**) at the end of our protocol. In CD45RA^+^ fraction, around 35% of TLs expressed CD62L, corresponding to TLs with T_SCM_ features (around 30% of total TLs). In CD45RA^-^ fraction, around 50% expressed CD62L, corresponding to TLs with T_CM_ features (around 10% of total TLs). Finally, in CD45RA^+^ and CD45RA^-^ fractions, CD62L^-^ M1-CTLs corresponded to TLs with T_EM_ features (around 60% of total TLs). There were no significant differences between patients and healthy donors (**Figures 4C, D**).

Since long-lived CTLs expressing CD62L have been shown to express high levels of CD27, CD28 and CD127 molecules (31), we compared the expression of these markers in CD62L^+^ and CD62L^-^ populations obtained with our AAPCs (**Figure 4E**). Importantly, we found that CD62L^+^ CTLs significantly expressed higher levels of CD28 and CD127 compared to their CD62L^-^ counterpart. The level of expression of CD27 was equally high in both populations (**Figure 4E**). As the vast majority of M1-CTLs in our experiments expressed both CD45RA and CD95 markers, we could conclude that M1-CTLs with a T_SCM_ phenotype according to CD95, CD45RA and CD62L expression indeed expressed high levels of CD27, CD28 and CD127.

In conclusion, by using our AAPC system, our protocol favored the rapid and efficient generation of antigen-specific TLs with long-lived and “young” phenotype.

### High Proliferative Capacity, Self-Renewal, and Multipotency *In Vitro* of Fluorescence-Activated Cell Sorting-Purified M1-CTLs With T_SCM_ Phenotype Features

**Figure 5:**
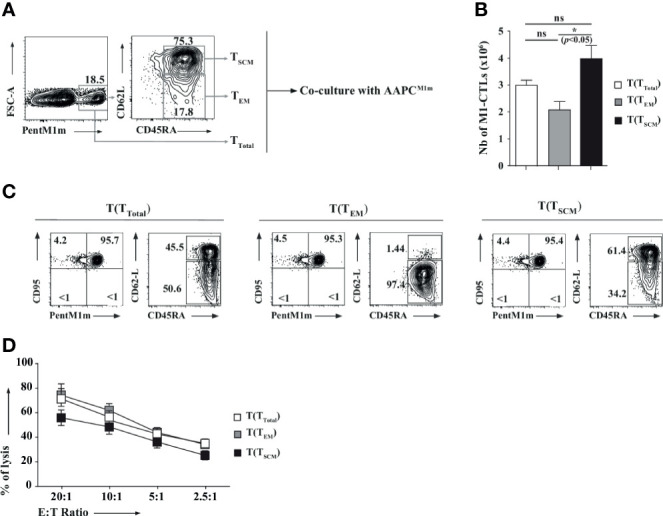
T lymphocytes with stem cell-like and effector memory phenotypes carry different proliferative, differentiation and functional capacities. **(A)** Total CTLs obtained at D21 were stained with PentM1m (left panel). Purified M1-CTLs were used in T_Total_ condition. Fluorescence-Activated Cell Sorting (FACS)-purified CD45RA^+^CD62L^+^ and CD45RA^+^CD62L^-^ M1-CTLs were described as T_SCM_ and T_EM_ respectively (middle panel) and amplified with AAPC^M1m^ for 14 days. **(B)** M1-CTL absolute numbers obtained at the end of the co-culture (D35) from M1-CTL-derived T_Total_ (T(T_Total_), white histogram), T_EM_ (T(T_EM_), grey histogram) and T_SCM_ (T(T_SCM_), black histogram) are represented. Histograms were obtained from three healthy donors and shown with standard errors of means. Statistical tests (*t* tests) were performed to compare both groups. Not significant (ns): *p* > 0.05, *p < 0.05. **(C)** Phenotype of T(T_Total_), T(T_EM_), and T(T_SCM_) M1-CTLs. Relative percentages in a representative experiment are represented in each relevant quadrant. **(D)** M1-CTL cytotoxicity was assessed in standard 4-hour ^51^Cr release assays. Target cells were T2 cells pulsed with 10µM of MART-1_A27L_ analogue peptide (M1m) or Flu Matrix Protein-derived peptide (FMP). Non-specific lysis was always lower than 10% and removed to obtain the specific lysis. The graph represents the results obtained with four healthy donors, each donor having been studied in three independent experiments. Data are represented with standard errors of means. Statistical tests (Friedman tests with Dunn’s post-tests) were performed to compare the different groups.

T_SCM_ have recently been described as memory TLs with high capacities to proliferate, and both to renew and to differentiate into other memory subpopulations (31, 32). Therefore, to further characterize the M1-CTLs displaying T_SCM_ phenotype features (CD95^+^CD45RA^+^CD62L^+^) in our co-cultures, all cells being CD95+, obtained TLs were sorted according to PentM1m, CD45RA and CD62L staining to separate M1-CTLs into “T_SCM_” (CD45RA^+^CD62L^+^) and “T_EM_” (CD45RA^+/-^CD62L^-^, **Figure 5A**), the main obtained populations. The purity of each population was always greater than 98% (data not shown). After 14 days of co-culture, expansion, phenotype and function of obtained “total”-, “T_EM_”- and “T_SCM_”-derived CTLs were studied. Interestingly, we observed around a 2-fold higher number of M1-CTLs derived from purified “T_SCM_” [T(T_SCM_)] compared to purified “T_EM_”-derived ones (T(T_EM_), **Figure 5B**). Phenotypic analysis of T(T_EM_) CTLs revealed stable lower expression of CD62L (**Figure 5C**, left panel) than in both T(T_SCM_) and T(T_Total_) populations, where a fraction of M1-CTLs maintained high expression of CD62L (**Figure 5C**, middle and right panels respectively). Interestingly, cells in both T(T_SCM_) and T(T_Total_) populations could differentiate into T_EM_ (**Figure 5C**, middle and right panels respectively). Yet, Chromium release assays showed that T(T_EM_) and T(T_Total_) CTLs displayed slightly higher cytotoxic capacities than T(T_SCM_) CTLs, less rich in functional T_EM_ cells (**Figure 5D**).

In conclusion, by using our AAPC system, obtained M1-CTLs with T_SCM_ phenotype features (CD95^+^CD45RA^+^CD62L^+^) displayed high proliferative capacity, self-renewal and multipotency *in vitro*.

## Discussion

In this study, we describe, for the first time to our knowledge, an efficient preclinical protocol, based on the use of artificial antigen presenting cells (AAPCs), allowing the expansion of anti-tumor specific CTLs with stem cell-like memory features. Our “off-the-shelf” NIH/3T3 fibroblast-derived AAPCs are easily generated, rapidly expanded and could express any HLA molecule and antigen of interest. Noteworthy, NIH/3T3 fibroblast-based protocols are already used in clinics with no reported adverse effect due to xenoreactivity (37). Moreover, to be fully cautious, in our protocol, AAPCs are irradiated (they are totally cleared off anyway after TL activation) and specific CTLs are very highly purified (CTLs of unknown specificity could decrease ACT efficiency and even have deleterious effects). GMP grade NIH/3T3-based AAPCs have already been constructed and their use is now envisaged in different severe infections and cancers.

In this study, we chose to use peripheral blood TLs (PBTLs). Many studies have already shown that peripheral blood is a good source of antigen-specific TLs (4, 14, 15, 17). PBTLs are easier to manipulate and less differentiated than tumor-infiltrating TLs.

The vast majority of M1-CTLs in the peripheral blood, not only of healthy donors but also of melanoma patients, even harbor the phenotype of naïve TLs (38). After stimulation, naïve TLs can indeed differentiate into different subsets of efficient long-lived memory TLs (31, 32).

Using our AAPC^M1m^, we efficiently stimulated functional TLs in melanoma patients and in healthy donors. Significantly higher M1-CTL percentages and absolute numbers were detected at D0 in the peripheral blood of melanoma patients, certainly due to the ongoing anti-tumor natural immune response. But these patients’ immune response might be negatively regulated because of tumor escape mechanisms, health problems or immunosuppressive treatments, which could affect M1-CTL initial proliferative capacities and explain M1-CTL lower enrichment in some patients. Nevertheless, our results indicate that patients’ peripheral blood M1-CTLs derived from patients’ PBTLs globally preserve high *in vitro* proliferative capacities and functions after two AAPC stimulations, which might not be the case for M1-CTLs derived from TILs because of TIL full differentiation and intra-tumoral strong immune escape mechanisms. After a short time of co-culture, corresponding to only two rounds of stimulation, allowing limited CTL manipulations, we always obtained pure M1-CTL numbers compatible with ACT (10^8^ to 10^9^ pure M1-CTLs obtained from 100ml of peripheral blood) even when a low frequency of M1-CTLs (lower than 0.05%) was detected in the peripheral blood (**Table 1** and data not shown).

Purified M1-CTLs displayed a rather large TCR diversity revealed by a repertoire study which could be even more extensive before clinical application. Interestingly, all amplified Vβ families have already been described after MART-1-specific CTL activation using autologous or allogeneic systems, indicating that our AAPC-based protocol did not induce any major bias in TL repertoire (4). Moreover, large TCR diversity has already been shown to be associated with better clinical outcome in melanoma patients (39, 40).

Efficient ACT requires sufficient number of TLs with strong functional capacities. We obtained with our protocol numerous pure M1-CTLs which were producing high amounts of IFNγ, TNFα and IL-2, and were able to specifically lyse both MART-1-pulsed T2 cells and melanoma-derived cell lines very efficiently, with a high functional avidity.

Efficient ACT also requires long-lived non-terminally differentiated CTLs for long term effects *in vivo*. We obtained with our protocol numerous pure M1-CTLs which expressed different long-lived TL markers (CD45RA, CD62L, CCR7, CD27, CD28 and Bcl-2) and no co-inhibitory or finally differentiated markers (CD57, CTLA4, PD-1 and FasL), PD-1 having recently been described as expressed by CD8+ progenitors giving rise to dysfunctional, exhausted-like TLs (41). Moreover, part of obtained M1-CTLs expressed skin homing factors (CLA, CCR4 and CCR10). All these phenotypic features could be of great interest for melanoma immunotherapy.

The subpopulation of memory TLs expressing CD95, CD45RA and CD62L in our study harbors stem cell-like memory TL (T_SCM_) features, as shown after cell sorting in **Figure 5**. These TLs display high proliferation, self-renewal and multipotency potentials. This has already been reported *in vivo* in nonhuman primates, in immunocompetent mice, and in immunodeficient mice with gene-modified human anti-tumor TLs (31–33). In the latter mouse models, all injected human T cells were gene-modified, in order to get *in vitro* sufficient numbers of tumor-specific cells with T_SCM_ features. Moreover, these cells differentiated *in vivo* into effector T cells, without retaining self-renewal capacities. This could be due, at least in part, to the absence of expression, in these mice, of the natural human ligands of CCR7 and CD62L expressed by human T cells with T_SCM_ features, leading to the absence of preferential migration of these cells into secondary lymphoid tissues (31, 32). To our knowledge, there is no available humanized mouse model yet (e.g. immunodeficient knock-in mice expressing human CCR7 and CD62L ligands in lymphoid tissues) allowing the *in vivo* thorough study of human T cells with T_SCM_ features. Nevertheless, recent studies have proven that these T cells were indeed associated with long term protection in humans (42, 43).

Obtaining in a reproducible manner stem cell-like memory T cells *in vitro* for ACT, without any TL gene modification, remains a challenge (34).

All published relevant methods, to our knowledge, aiming at amplifying *in vitro* tumor-specific CTLs, including MART-1-specific T cells, are based on the use of autologous antigen presenting cells, allogeneic cells, K562-derived antigen presenting cells or anti-CD3/anti-CD28 beads (14, 17, 44–49). They all require multiple stimulations for obtaining large numbers of specific CTLs and lead to the expansion of central or effector memory T cells (8, 50). Indeed, to our knowledge, a clinical protocol to expand numerous antigen-specific CTLs with T_SCM_ features and without TL gene modification does not exist yet.

Interestingly, TL stimulation with our NIH/3T3-derived AAPC-based protocol allowed the generation of CTLs with not only T_CM_ and T_EM_ but also T_SCM_ features in an antigen-specific context from PBTLs of all tested patients and healthy donors. Together, the different CTL populations we obtained in large numbers with our protocol could greatly improve the anti-tumor response, allowing short and long-term effects after CTL injection.

Surprisingly, IL-7 or IL-15, known to promote stem cell-like memory T cells (51), alone or in all different combinations with or without IL-2 in our co-cultures, did not lead to greater numbers of T cells with T_SCM_ features. IL-7 maintained the percentage of T cells with T_SCM_ features without leading to cell expansion. IL-15 maintained the percentage of T cells with T_SCM_ features and led to their expansion, but in a way comparable to IL-2 (**Supplementary Material**).

Altogether, our results are probably due, at least in part, to: (i) the particular immunological synapse created between specific CTLs and our NIH/3T3 fibroblast-derived AAPCs (expressing only potent accessory molecules at a high level), (ii) the few TL manipulations that our AAPC-based protocol requires (with only two stimulations in 35 days) and (iii) the low doses of IL-2 we added to our co-cultures, which did not trigger too strong TL activation (28).

MART-1 is a particular antigen. In the peripheral blood of melanoma patients and of healthy donors, M1-CTLs can be found at a higher frequency than other tumor-associated antigen (TAA)-specific CTLs and these CTLs can more easily be expanded *in vitro* (52). Still, the study of M1-CTLs is highly relevant since MART-1 is over-expressed in many melanoma and M1-CTLs have been used in many clinical trials. Our AAPCs have already been described as capable of expanding functional specific CTLs against other virus- and tumor-associated antigens such as pp65, gp100 and hTERT (15, 53). Many TAA-specific CTLs, other than M1-CTLs, harboring a naïve phenotype, have been found, although at a lower frequency than M1-CTLs in the peripheral blood of healthy donors and cancer patients (54–56). Therefore, it should be possible to obtain T cells with T_SCM_ features for these antigens, and it will be important in the near future to test this hypothesis in order to broaden the interest of our protocol.

In conclusion, we describe a novel and unique AAPC-based protocol allowing the activation and the expansion, in a specific manner, of sufficient numbers of highly functional anti-melanoma CTLs with a suitable phenotype for immunotherapy, i.e. not only with T_EM_ and T_CM_ but also with T_SCM_ features, both in patients and in healthy donors, which could allow short and long term anti-tumor responses *in vivo*. We now intend to use GMP grade AAPCs and optimized CTL purification procedure in our protocol and to confirm these *in vitro* results in a relevant animal model, in order to be able to propose a clinical trial based on the use of these AAPCs for melanoma patients.

## Data Availability Statement

The original contributions presented in the study are included in the article/**Supplementary Material**. Further inquiries can be directed to the corresponding author.

## Ethics Statement

The studies involving human participants were reviewed and approved by Rouen University Hospital Institutional Review Board “Comité de Protection des Personnes Nord-Ouest I”. The patients/participants provided their written informed consent to participate in this study.

## Author Contributions

MH and J-FC performed the experiments, analyzed the data, and wrote the paper. ED, FB, EF, PM, and AD helped to perform experiments. A-BD-M provided clinical data and samples. SA and OB helped to design the study and to write the paper. J-BL designed and supervised the study, analyzed the data, and corrected the paper. All authors contributed to the article and approved the submitted version.

## Funding

MH and J-FC’s PhD fellowships were provided by “La Ligue Contre le Cancer de Seine-Maritime”, France. This study was supported by “La Ligue Contre le Cancer de Haute-Normandie”, France.

## Conflict of Interest

The authors declare that the research was conducted in the absence of any commercial or financial relationships that could be construed as a potential conflict of interest.

## Publisher’s Note

All claims expressed in this article are solely those of the authors and do not necessarily represent those of their affiliated organizations, or those of the publisher, the editors and the reviewers. Any product that may be evaluated in this article, or claim that may be made by its manufacturer, is not guaranteed or endorsed by the publisher.
