# Supplementary material for: Generation of Pure Highly Functional Human Anti-Tumor Specific Cytotoxic T Lymphocytes With Stem Cell-Like Memory Features for Melanoma Immunotherapy
Source: Front Immunol. 2021 Sep 8;12:674276. doi: 10.3389/fimmu.2021.674276 (PMC8456028; doi:10.3389/fimmu.2021.674276)
Supplement: Supplementary file 1 [file DataSheet_1.pdf]

Supplementary Material

Supplementary Materials and Methods

Stimulation of T lymphocytes (TLs) with different cytokines

PBMCs were collected by density centrifugation on a lymphocyte separation medium (Eurobio, Courtaboeuf, France). Irradiated $AAPC^{M1m}$ (10^5 cells, 25Gy) were plated in 24-well plates in AIM-V medium (Invitrogen, Saint Aubin, France) supplemented with 5% DCS. The next day, total TLs were negatively selected from PBMCs using Dynabeads untouched human T cell Kit (Invitrogen) according to the manufacturer's instructions. Next, purified TLs (10^6 cells) resuspended in AIM-V medium were co-cultured with irradiated $AAPC^{M1m}$ for 21 days as previously described [1, 2]. On day 7 of the co-culture and then, every third day, 20 IU/ml of IL-2, 10 ng/ml of IL-7 and/or 10 ng of IL-15 (R&D systems, Abington, UK) were added to the wells. At D21, without sorting of specific M1-CTLs, TLs ($0.5x10^6$) were amplified on 10^5 irradiated $AAPC^{M1m}$ for an additional 14 days as described above except that IL-2, IL-7 and /or IL-15 were first added 2h after co-culture start.

Supplementary Figure

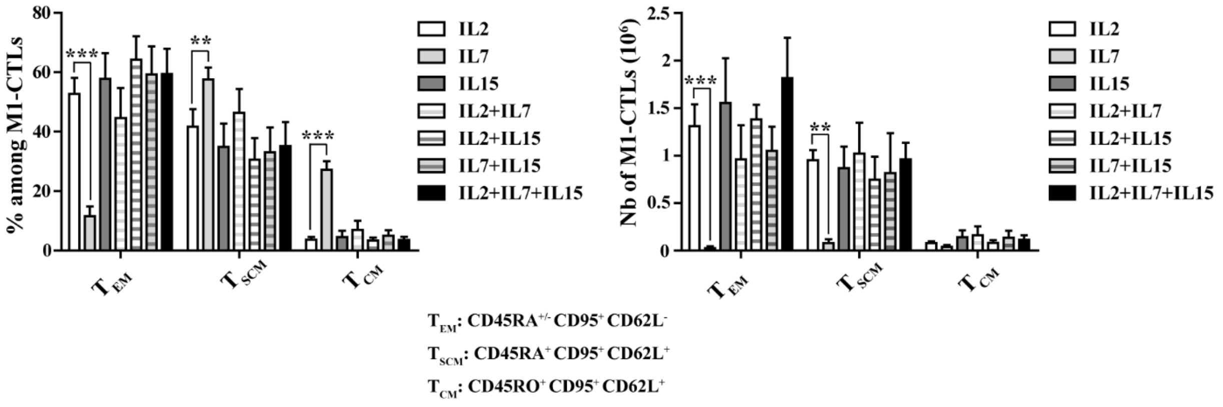

Supplementary Figure 1. Effects of different cytokines on M1-CTLs obtained at the end of our AAPC-based protocol. M1-CTL percentages (left panel) and absolute numbers (right panel) of the three memory T cell subsets defined according to the expression of CD45RA, CD45RO, CD95 and CD62L [effector memory (EM) T cells, T cells with stem cell-like memory (SCM) features and central memory (CM) T cells] are represented on histograms. M1-CTLs were obtained as described under Supplementary Materials and Methods after stimulation in the presence of IL-2 (20 UI/ml), IL-7 (10 ng/ml) and IL-15 (10 ng/ml), alone or in combination, for six healthy donors, and data are shown with standard errors of means. Statistical tests (Two Way ANOVA test with Bonferroni's post-test or Friedman test with Dunn's post-test) were performed to compare the different groups. **: $p<0.01$, ***: $p<0.001$

Supplementary References

1. Latouche JB, Sadelain M. (2000) Induction of human cytotoxic T lymphocytes by artificial antigen-presenting cells. Nat Biotechnol 18: 405–409. doi: 10.1038/74455.
2. Fauquembergue E, Toutirais O, Tougeron D, Drouet A, Le Gallo M, Desille M, et al. (2010) HLA-A*0201-restricted CEA-derived peptide CAP1 is not a suitable target for T-cell-based immunotherapy. J Immunother 33: 402–413. doi: 10.1097/CJI.0b013e3181d366da.
